# Phytochemical Profiling and Antibacterial Activity of Moroccan Monofloral Honeys Against Multidrug‐Resistant Uropathogens: In Vitro, ADMET, Molecular Docking, and Molecular Dynamics Approaches

**DOI:** 10.1002/fsn3.72165

**Published:** 2026-08-27

**Authors:** Mourad Chikhaoui, Aziz Galman, Ahmed Chetoui, Oussama Khibech, Mohamed Reda Kachmar, Toufik Bouddine, Mohamed Bouhrim, Fahd A. Nasr, Mohammed Al‐Zharani, Ashraf Ahmed Qurtam, Rachid Lotfi, Houda El Hajjouji

**Affiliations:** ^1^ Ecology and Environment Laboratory, Faculty of Sciences Ben M'sik Hassan II University Casablanca Morocco; ^2^ Higher Institute of Nursing Professions and Health Techniques Beni‐Mellal Morocco; ^3^ Applied Engineering and Technology Laboratory, Faculty of Sciences, and Techniques Sultan Moulay Slimane University Beni‐Mellal Morocco; ^4^ Biological Engineering Laboratory, Faculty of Sciences, and Techniques Sultan Moulay Slimane University Beni‐Mellal Morocco; ^5^ Applied and Environmental Chemistry Laboratory, Department of Chemistry, Faculty of Sciences Mohammed I University Oujda Morocco; ^6^ Valorization of Medicinal and Aromatic Plants and Environment Team, Faculty of Sciences Moulay Ismail University Meknes Morocco; ^7^ Bioactive and Environmental Health Laboratory, Faculty of Sciences Moulay Ismail University Meknes Morocco; ^8^ Laboratoires TBC, Laboratory of Pharmacology, Pharmacokinetics, and Clinical Pharmacy Faculty of Pharmaceutical and Biological Sciences Lille France; ^9^ Biology Department, College of Science Imam Mohammad Ibn Saud Islamic University (IMSIU) Riyadh Saudi Arabia

**Keywords:** antibacterial activity, computational biology, monofloral honey, phenolic compounds

## Abstract

Urinary tract infections represent a significant public health issue exacerbated by bacterial antibiotic resistance. Honey, due to its phenolic compounds, offers a promising natural alternative. This study explores the phenolic profile of monofloral honeys from the Beni Mellal‐Khenifra region and their antibacterial activity against multidrug‐resistant strains. Four monofloral honeys from Beni Mellal‐Khenifra were analyzed for their phenolic composition and evaluated for antibacterial activity against multidrug‐resistant uropathogenic bacteria. Computational studies were also performed to explore the potential mechanisms of action of the identified compounds. HPLC‐UV detection identified distinct phenolic profiles in four monofloral honeys. Carob honey showed the highest diversity, with eight compounds identified, notably myricetin (63.99 μg/mg), trans‐ferulic acid (33.99 μg/mg), gallic acid (21.02 μg/mg), naringin (17.95 μg/mg), catechin (3.66 μg/mg), and trans‐cinnamic acid (2.75 μg/mg). Euphorbia honey was rich in hydroxycinnamic acids, especially caffeic acid (285.35 μg/mg), p‐coumaric acid (190.93 μg/mg), and trans‐ferulic acid (125.37 μg/mg). Anise honey contained high levels of naringin (669.84 μg/mg), along with myricetin (90.08 μg/mg) and 3‐hydroxybenzoic acid (76.59 μg/mg). In contrast, citrus honey exhibited the lowest polyphenolic content, with only gallic acid (0.21 μg/mg) and catechin (2.69 μg/mg) detected. The honey samples exhibited notable antibacterial activity against multidrug‐resistant uropathogenic bacteria, including *E. coli, K. pneumoniae*, and *S. aureus* . Among them, euphorbia and carob honeys demonstrated the highest antibacterial efficacy, with MBC values of 25% (v/v) against *S. aureus* and 50% against *E. coli* and *K. pneumoniae* . In contrast, citrus and anise honeys showed lower bactericidal activity, with MBC values reaching 75% (v/v) for *E. coli* and *K. pneumoniae* , and 50% for *S. aureus* . *In silico,* analyses showed that kaempferol, quercetin, catechin, and myricetin exhibit high binding affinities (up to −8.3 kcal/mol) against major bacterial targets. Molecular dynamics confirmed the stability of the quercetin‐GyrB complex (RMSD ~0.35 nm). ADMET and toxicity profiles identified catechin and 3‐hydroxybenzoic acid as the most promising candidates, combining good intestinal absorption, low predicted toxicity (LD50≈10,000 mg/kg), and favorable pharmacokinetics. These findings confirm the important role of honey's phenolic compounds in inhibiting multidrug‐resistant bacteria. Future research should focus on in vivo validation and the formulation of effective therapeutic products based on these honeys.

## Graphical abstract

Multidrug‐resistant bacteria infections require alternative antimicrobial strategies. This study evaluated the phenolic composition, antibacterial activity, and *in silico* properties of four Moroccan monofloral honeys. Carob honey showed the greatest phenolic diversity, whereas citrus honey contained the lowest polyphenolic content. Euphorbia and carob honeys exhibited the strongest antibacterial activity against *E. coli, K. pneumoniae*, and *S. aureus* , while citrus and anise honeys were less effective. Molecular docking revealed that kaempferol, quercetin, catechin, and myricetin displayed high binding affinities against key bacterial targets. ADMET analysis identified catechin and 3‐hydroxybenzoic acid as the most promising compounds, with favorable intestinal absorption and safety profiles.

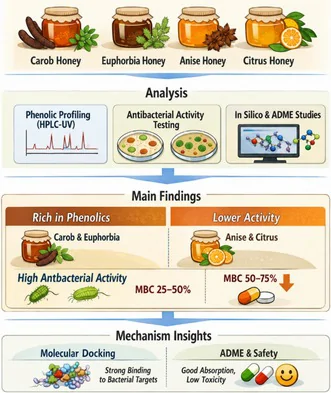

## 1 Introduction

Urinary tract infections (UTIs) are a common reason for medical consultations and antibiotic prescriptions in general practice. They represent the second most frequent site of community‐acquired bacterial infections after respiratory infections and remain the leading cause of reported nosocomial infections (Thiolet et al. 2007), thus posing a major public health concern. Antibiotic susceptibility testing demonstrated that the majority of isolates responsible for UTIs exhibited high levels of resistance to the most commonly used antibiotics (Chikhaoui et al. 2023). The growing antibiotic resistance among bacteria involved in UTIs limits the choice of available antibiotics, highlighting the crucial importance of laboratory tests both for the accurate diagnosis of these infections and for guiding appropriate antibiotic therapy. However, the excessive and sometimes unfounded use of antibiotics to treat UTIs is a problematic practice. It is concerning to note that more than 50% of antibiotics prescribed for empirical treatment are deemed inappropriate (Dellit et al. 2007). This excessive and non‐targeted use of antibiotics exposes patients to an increased risk of toxicity while fueling the development of bacterial resistance to medications. Furthermore, the emergence of resistance in nearly all potentially pathogenic bacteria is alarming, making the treatment of infections increasingly difficult, or even impossible in some cases (Carle 2009). Consequently, the World Health Organization (WHO) has warned that antibiotic resistance could become the leading cause of mortality worldwide by 2050 (Bertholom 2016). To address this crisis, the WHO has strongly encouraged member states to explore alternative solutions, including natural product‐based alternative medicines (Mama et al. 2019). This alternative approach could offer new perspectives in infection treatment while reducing excessive antibiotic use and potentially slowing the spread of bacterial resistance. Among the natural products attracting particular interest in medical research, honey holds a notable place. It has been used as a natural remedy since 2000 bc (Ballot‐Flurin 2013; Johnston et al. 2018). However, its antibacterial properties were not scientifically demonstrated until 1892 (Mama et al. 2019). This discovery paved the way for numerous subsequent studies that confirmed honey's effectiveness against a wide range of bacterial strains, including aerobic, anaerobic, Gram‐positive, and Gram‐negative bacteria (Belhaj et al. 2016; Bucekova et al. 2019; Elbanna et al. 2014; Hegazi et al. 2017; Montenegro et al. 2009). Encouragingly, honey has also shown promising results in combating antibiotic‐resistant bacteria (Agbabiaka et al. 2020; Farhana et al. 2023; McLoone et al. 2020; Owayss et al. 2020). Beyond its antibacterial properties, honey offers several additional advantages, including its non‐toxic nature, minimal risk of adverse effects, the absence of bacterial resistance development, relatively low maintenance costs, and widespread local availability (Farhana et al. 2023; Majtan et al. 2020). The antibacterial properties of honey vary significantly depending on its physicochemical characteristics, such as acidity, osmolality levels, and enzymatic generation of hydrogen peroxide (H_2_O_2_) (Hegazi et al. 2017). Moreover, the presence of certain proteinaceous or phytochemical compounds, such as aromatic acids and phenolic compounds, can also influence these antibacterial properties (Cheng et al. 2019). In traditional medicine, the use of honey as a treatment has been reported as an old practice dating back centuries in countries like China, India, Egypt, Greece, and Rome, and has been documented in several religious texts, including the Quran, Veda and the Bible (Dai et al. 2021; Hellner et al. 2008; Shahsuvaryan 2016). Several in vitro and in vivo studies have demonstrated the antimicrobial, antiviral, antifungal, anticancer, and antidiabetic activity of honey (Afrin et al. 2018; Cucu et al. 2021; Mellor et al. 2010; Pichichero et al. 2010; Premratanachai and Chanchao 2014; Samarghandian et al. 2011). In addition, protective effects on the cardiovascular, nervous, respiratory, and gastrointestinal systems have been reported (Alvarez‐Suarez et al. 2016). Moreover, a protective effect of honey has also been observed under physiological conditions characterized by high levels of free radicals, such as those of athletes practicing different sports (Peternelj and Coombes 2011). In Morocco, honey constitutes an essential component of both food and traditional medicine (Chikhaoui et al. 2023). Consequently, its per capita consumption (0.52 to 0.75 kg/year) is relatively high compared to neighboring countries such as Algeria (0.20 kg/year) and Tunisia (0.17 to 0.20 kg/year) (Haderbache and Mohammedi 2015; Hussein 2000; Moujanni et al. 2017). According to the Haut‐Commissariat au Plan, the Beni Mellal‐Khenifra region ranks third nationally in honey production (HCP 2017). This production includes honeys with distinctive biological, palynological, and physicochemical properties, which support their use not only as natural sweeteners but also as preventive agents or even as therapeutic remedies (Belhaj et al. 2016). A study we conducted in the Beni Mellal‐Khenifra region revealed that 82% of the people surveyed used honey to treat a total of 42 symptoms or conditions. The most frequently mentioned ailments were respiratory diseases, skin conditions, and gastrointestinal disorders (Chikhaoui et al. 2023). However, despite the widespread medicinal use of honey, no in‐depth scientific study has been conducted in this region to characterize its bioactive compounds or evaluate its therapeutic potential. It is within this context that the present research aims to characterize the phenolic profile of monofloral honeys from the Beni Mellal‐Khenifra region (Morocco) and to assess their antibacterial activity against multidrug‐resistant uropathogenic strains.

## 2 Materials and Methods

### 2.1 Chemicals and Reagents

For chromatographic analyses, acetonitrile (HPLC grade) and formic acid (≥ 98%) used in the mobile phase were purchased from Sigma‐Aldrich (USA). Ultrapure water was produced using a Millipore purification system. Phenolic acid and flavonoid standards, including gallic acid, caffeic acid, p‐coumaric acid, trans‐ferulic acid, quercetin, kaempferol, myricetin, naringin, and catechin, used for compound identification and quantification, were obtained from Macherey‐Nagel (Düren, Germany). For microbiological assays, culture media such as Mueller‐Hinton broth, Eosin Methylene Blue (EMB) agar, and Baird‐Parker agar were supplied by Biokar Diagnostics (France). Biochemical reagents required for bacterial strain identification, including Indole, Methyl Red, Voges‐Proskauer, Citrate, Catalase, and Coagulase tests, were provided by BioMérieux. Regarding in silico analyses, molecular structures were drawn using ChemDraw 22 (PerkinElmer). Molecular docking was performed with PyRx 0.9.9 using AutoDock Vina 1.2.5, and molecular dynamics simulations were conducted with GROMACS 2021.3. The three‐dimensional structures of target proteins were retrieved from the Protein Data Bank (PDB). Pharmacokinetic and toxicity evaluations of the compounds were carried out using the SwissADME, ADMETlab 3.0, and ProTox‐III online platforms. Chromatographic data acquisition and processing were performed using ChromNAV 2.0 software (JASCO Corporation, Japan).

### 2.2 Honey Sample Collection

This study focused on four honey samples from four different sites (Tadla, Tagzirte, El Ksiba and Souk Sebt) in the Beni Mellal region, specifically citrus, euphorbia, anis, and carob honeys.

The identification of monofloral honeys was not based on pollen analysis but on a controlled field collection strategy. Empty frames were initially placed in hives located in areas dominated by a single plant species (carob, citrus, euphorbia, and anise) during the corresponding flowering period. Honey was harvested exclusively from these frames, ensuring the predominant botanical origin of the samples, in accordance with common beekeeping practices when pollen analysis is not performed.

The honeys were collected between March and October 2021 (Table 1). All samples were filtered through sterile gauze to remove debris and stored in sterile airtight glass containers at 4°C in the dark until use (Bogdanov and Blumer 2001).

**TABLE 1:** Botanical‐geographical origins and harvesting periods of the studied honey samples.

Honey samples	Botanical origins	Harvesting site	Geographical coordinates			Harvesting period
			Latitude (N)	Longitude (W)	Altitude (m a.s.l.)	
Citrus honey	*Citrus*	Tadla	30°15′11.0919″	6°35′11.5458″	548	March 2021
Euphorbia honey	*Euphorbia*	Tagzirt	32°26′51.6951″	6°10′6.64932″	737	July 2021
Carob honey	*Ceratonia siliqua*	El Ksiba	32°35′16.3644″	6°3′49.15152″	765	October 2021
Anise honey	*Pimpinella anisum*	Souk Sebt	32°15′31.1281″	6°49′29.3458″	443	October 2021

*Note:* m a.s.l.: meters above sea level.

### 2.3 Preparation of Honey Solutions

In this study, different concentrations (25%, 50%, 75%, and 100%) of each honey sample were prepared. To prepare a 75% honey solution (v/v). Similarly, to prepare 50% and 25% honey solutions (v/v), 0.5 mL of honey was diluted in 0.5 mL of sterile water, and 0.25 mL of honey was diluted in 0.75 mL of sterile water, respectively (Mama et al. 2019).

### 2.4 Phytochemical Characterization

#### 2.4.1 Determination of Total Phenol Content (TPC)

The TPC was determined using the Folin–Ciocalteu reagent method as described by Živić et al. (2019) with some modifications. A volume of 0.2 mL of honey dissolved in distilled water was mixed with 1.5 mL of Folin–Ciocalteu reagent (10%) diluted in ultrapure water. After 5 min, 1.5 mL of sodium carbonate (Na_2_CO_3_; 7.5%) dissolved in ultrapure water was added to the mixture, and the final solution was incubated in the dark for 30 min at 25°C. Absorbance was measured at 760 nm using a spectrophotometer, and TPC was calculated based on a standard calibration curve using gallic acid, expressed as milligrams Gallic Acid Equivalent per 100 g of honey (mg GAE/100 g). The standard curve was produced for gallic acid within the concentration range from 100 to 1000 mg/L (*R* ^2^ = 0.9998, y=0.1254x−0.1424). Three independent experiments were performed in triplicate, and results were reported as the mean ± standard error.

#### 2.4.2 Determination of Total Flavonoids Content (TFC)

The TFC was determined using the method described by Miguel et al. (2010), with slight modifications. Briefly, 2.5 mL of aqueous extracts obtained from the studied honey samples, previously dissolved in ultrapure water, were mixed with 2.5 mL of a 2% aluminum chloride (AlCl_3_) solution prepared in ultrapure water. After incubation for 1 h at room temperature, absorbance was measured at a wavelength of 420 nm using a spectrophotometer. Quercetin was used as the reference standard to construct a calibration curve under the same experimental conditions. The flavonoid content was expressed as milligrams of quercetin equivalent per 100 g of crude extract (mg QE/100 g). Each experiment was conducted in triplicate, with results presented as the mean ± standard error.

#### 2.4.3 Identification of Phenolic Compounds

The phenolic composition of the honey samples was identified using reverse‐phase high‐performance liquid chromatography (RP‐HPLC), following the method previously described by Kachmar et al. (2025). The column used was an EC NUCLEOSIL (5 μm, C18, 100–5, 250 × 4.6 mm; Macherey‐Nagel, Germany) and the conditions were as follows. The mobile phase consisted of two solvents: water‐formic acid (1%) (A) and acetonitrile (B), starting with 5% B and using a gradient to obtain 15% B at 3 min, 25% B at 13 min, 30% B at 25 min, 35% B at 35 min, 45% B at 39 min, 55% B at 47 min, 75% B at 56 min and 100% B at 60 min. The UV detection was at 280 nm. The injection volume was 20 μL, and the flow rate was fixed at 0.9 mL/min during the whole experiment. The HPLC system was controlled by ChromNAV 2.0—JASCO software. Identification was performed by comparing the retention time of each peak in the honey chromatogram with the corresponding standard. Moreover, to ensure the superposition of each standard with the corresponding molecule, each extract was spiked with standards corresponding to the suspected molecules, and an increase was observed in the concentration of these molecules.

#### 2.4.4 Quantification of Phenolic Compounds Contents

For the quantification of phenolic compounds, 20 μL of aqueous freeze‐dried extract dissolved in ultrapure water was analyzed using an HPLC‐UV unit, with an NUCLEOSIL EC column (5 μm, C18, 100–5, 250 x 4.6 mm; Macherey‐Nagel, Germany), and following the conditions described in the previous section. Spectral data of all peaks were collected at a wavelength λ = 280 nm. Data were processed on a system assisted with ChromNAV 2.0 HPLC software—JASCO. The concentrations of phenolic acids and flavonoids detected in the aqueous extracts of the studied honey extracts were deduced from the calibration curves established with the corresponding standards.

### 2.5 Antibacterial Activity Testing

#### 2.5.1 Collection of Bacterial Cultures

The in vitro antimicrobial activity of the honeys was tested against three human uropathogenic bacterial strains: *Escherichia coli* ( *E. coli* ), *Klebsiella pneumoniae* ( *K. pneumoniae* ), and *Staphylococcus aureus* ( *S. aureus* ), which were isolated from urine samples collected at the Clinical Laboratory of the Regional Hospital Center (CLRHC) of the Beni Mellal‐Khenifra region. The purity of strains was confirmed through subculturing on selective media, specifically Eosin Methylene Blue (EMB) agar for *E. coli* and *K. pneumoniae* , and Baird‐Parker agar for *S. aureus* . To accurately verify the identity of the organisms, a series of microbiological and biochemical tests were performed, including Gram staining, the Indole test, the Methyl Red test, the Voges‐Proskauer test, the Citrate test, the Catalase test, and the Coagulase test. The purified strains were subjected to an antibiogram test, then stored in a nutrient medium consisting of 2/3 Brain Heart Infusion (BHI) and 1/3 glycerol at −20°C for long‐term storage.

#### 2.5.2 Determination of the Minimum Inhibitory Concentration (MIC) and Minimum Bactericidal Concentration (MBC) of Honey Samples

The MIC of the honey samples was determined using the macrodilution tube method, also known as the turbidimetric assay (Mama et al. 2019; Fritsche et al. 2007). Mueller‐Hinton broth was prepared as the culture medium, and seven sterile test tubes, labeled 1 to 6, were used. Tube 1 served as a negative control to verify the absence of contamination, while tube 2 acted as a positive control to confirm normal bacterial growth. Tubes 3 to 6 contained progressively lower concentrations of honey at 100%, 75%, 50%, and 25% (*v/v*), respectively. Except for tube 1 (negative control), all other tubes were inoculated with 100 μL of a fresh bacterial suspension of the tested pathogenic bacteria, adjusted to a 0.5 McFarland standard (1 × 10^6^ CFU/mL). Following incubation for 24 h at 37°C under aerobic conditions, the tubes were visually examined, and the MIC was recorded as the lowest honey concentration in which microbial turbidity was not observed. To determine the MBC, samples from the tubes containing honey concentrations equal to or greater than the MIC were cultured onto sterile nutrient agar plates (EMB for *E. coli and K. pneumoniae*, and Baird‐Parker for *S. aureus* ) using the streak‐plate technique. The plates were then incubated for 24 h at 37°C under aerobic conditions. The lowest concentration of honey that showed no growth of the tested organisms was considered the MBC (Mama et al. 2019). All experiments were performed in triplicate, including three independent biological replicates, and each measurement was conducted in three technical replicates to ensure the reproducibility and reliability of the results.

### 2.6 In Silico Analysis

#### 2.6.1 Molecular Docking

Molecular docking was carried out in PyRx 0.9.9 running AutoDock Vina 1.2.5 (Trott and Olson 2010). The eleven study ligands and the reference ciprofloxacin were drawn in ChemDraw 22, exported as SDF files, energy‐minimized with the Universal Force Field, protonated, and converted to PDBQT directly within PyRx. The three target structures (PDB 1KZN, 1SHV, 3ZG5) were prepared in AutoDockTools 1.5.7 by removing crystallographic waters, adding polar hydrogens, and assigning Gasteiger charges. Grid boxes were then defined as follows: 1KZN center (18.38, 30.67, 36.62 Å) and dimensions 12.33 × 15.80 × 16.43 Å^3^; 1SHV center (30.30, 24.85, 4.088 Å) and dimensions 18.18 × 10.00 × 13.11 Å^3^; 3ZG5 center (3.59, −9.60, 59.44 Å) and dimensions 10.07 × 10.69 × 11.46 Å^3^. Redocking the native ligands at an exhaustiveness of 8 reproduced their experimental poses with RMSD values of 0.313 Å (1KZN), 1.462 Å (1SHV) and 1.117 Å (3ZG5), confirming protocol reliability. All other Vina parameters were left at default settings, and these validated grids were subsequently used to dock the full polyphenol set alongside ciprofloxacin.

#### 2.6.2 Molecular Dynamics Simulations

Molecular‐dynamics simulations were run in GROMACS 2021.3 (Abraham et al. 2015). Using gmx pdb2gmx with the AMBER99SB‐ILDN force field, the protein was first preprocessed to add missing hydrogens and assign the correct protonation states. Ligand parameters were generated separately, yielding validated.itp and .gro files that were then merged with the protein to assemble the full complex. The system was centered in a cubic TIP3P water box, neutralized with counter‐ions, and subjected to steepest‐descent energy minimization. Temperature and pressure were stabilized through successive NVT and NPT equilibration phases, after which a 100 ns production run was carried out. Coordinates and velocities were recorded at regular intervals, producing a trajectory that documents the complex's stability, conformational behavior, and key intermolecular interactions under near‐physiological conditions.

#### 2.6.3 In Silico Analysis of Pharmacokinetic Properties

Each molecule was first sketched in ChemDraw and converted to a canonical SMILES string, then processed by two prediction suites: SwissADME (Daina et al. 2017), which supplied the core physicochemical descriptors and drug‐likeness filters, and ADMETlab 3 (Fu et al. 2024), used here exclusively to estimate melting point, boiling point, plasma clearance, and half‐life (t_0.5_). Merging the SwissADME output with these four ADMETlab 3 parameters produced, for each compound, a concise ADME fingerprint that offers an early appraisal of its likely in vivo behavior.

#### 2.6.4 In Silico Prediction of Toxicity

Toxicity profiles were generated with ProTox‐III in line with current best‐practice guidelines (Banerjee et al. 2024). Validated ChemDraw SMILES strings were uploaded to the platform, which merges extensive toxicology databases with ensemble machine‐learning and Bayesian classifiers to flag structural alerts. For each molecule, ProTox‐III provides a complete endpoint panel, including acute oral LD_50_, its corresponding GHS hazard class, and percentage‐likelihood scores for hepatotoxicity, carcinogenicity, immunotoxicity, mutagenicity, and cytotoxicity, thereby highlighting potential in vivo liabilities. This rapid, high‐resolution screening streamlines candidate prioritization before laboratory testing and accelerates data‐driven decisions in drug‐discovery and environmental‐safety workflows.

### 2.7 Statistical Analysis

Statistical analyses were conducted using SPSS version 25.0. Descriptive statistics were calculated and presented as means ± standard deviations (SD). Differences in total polyphenol content among honey samples were evaluated using one‐way analysis of variance (ANOVA) followed by Tukey's post hoc test (*p* < 0.05). Before applying the tests, the assumptions of normality and homogeneity of variances were verified. Relationships between phytochemical parameters and the bactericidal activity of honey were evaluated using Pearson correlation coefficients (*r*).

## 3 Results and Discussion

### 3.1 Phytochemical Characterizations

#### 3.1.1 Polyphenol Content of Honey Extracts

**FIGURE 1:**
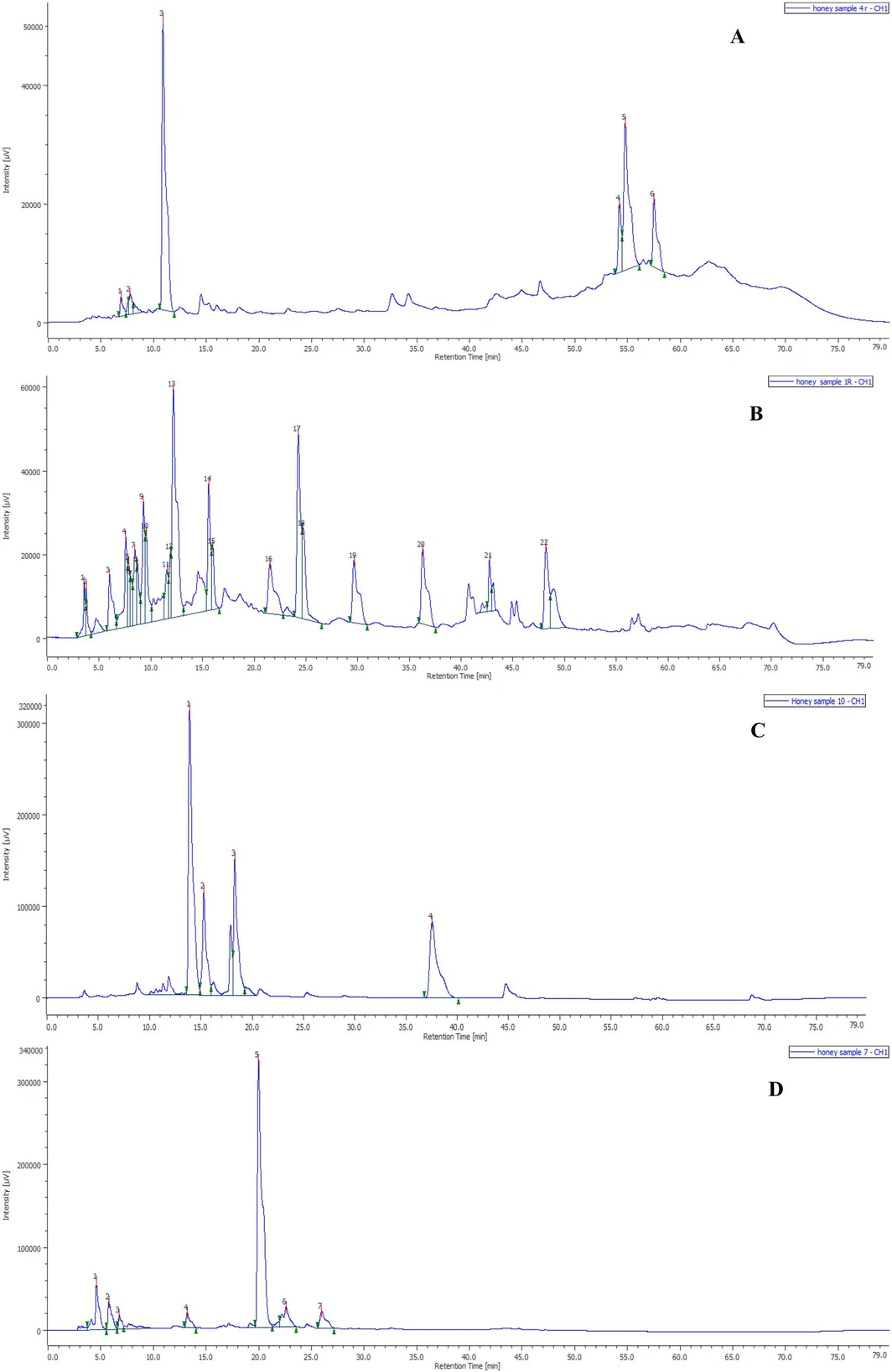
HPLC chromatograms of honey extracts at 280 nm (A) Citrus honey; (B) Carob honey; (C) Euphorbia honey; (D) Anise honey.

The analysis revealed distinct phenolic markers for each honey type, highlighting the significant influence of botanical origin and geographical factors on their chemical composition (Thiolet et al. 2007; Bucekova et al. 2019). The chromatographic conditions allowed for effective separation of the compounds, as shown in Figure 1. Carob honey exhibited the highest diversity of phenolic compounds, with a total of eight identified compounds, namely myricetin (63.99 μg/mg), trans‐ferulic acid (33.99 μg/mg), gallic acid (21.02 μg/mg), naringin (17.95 μg/mg), catechin (3.66 μg/mg), and trans‐cinnamic acid (2.75 μg/mg), kaempferol and quercetin (identified but not quantified). Furthermore, Euphorbia honey was found to contain four phenolic compounds and was notably characterized by a high concentration of hydroxycinnamic acids, particularly caffeic acid (285.35 μg/mg), p‐coumaric acid (190.93 μg/mg), and trans‐ferulic acid (125.37 μg/mg), which were either absent or present in much lower quantities in the other samples. Additionally, it contained a flavonoid (quercetin) which was not quantified. On the other hand, anise honey contained three phenolic compounds and exhibited exceptionally high levels of naringin (669.84 μg/mg), along with significant concentrations of myricetin (90.08 μg/mg) and 3‐hydroxybenzoic acid (76.59 μg/mg). In contrast, citrus honey exhibited only two phenolic compounds, gallic acid (0.21 μg/mg) and catechin (2.69 μg/mg), indicating a modest polyphenol content. Detailed retention times and concentrations of the identified compounds are presented in Table 2 and Figure 1. To the best of our knowledge, no prior studies have examined the phenolic composition of monofloral honeys from the Beni Mellal‐Khenifra region, highlighting the novelty and regional relevance of our findings. Nevertheless, the results of this study align with several international studies. For instance, research in New Zealand and Australia identified similar phenolic compounds, such as gallic, caffeic, and p‐coumaric acids, which we also detected (Kaškonienė et al. 2009). In Poland and Bangladesh, flavonoids like catechin, naringin, and myricetin were found, corroborating our findings in anise and euphorbia honeys (Moniruzzaman et al. 2014; Güneş et al. 2017). Additionally, studies in Algeria and Turkey identified compounds such as myricetin and ferulic acid, present in our citrus and carob honeys (Amessis‐Ouchemoukh et al. 2017; Isa et al. 2020). Despite geographical differences, the consistent presence of common phenolic compounds such as gallic acid, caffeic acid, and catechin across various studies highlights the conserved biosynthetic pathways in the plant species frequented by bees. These similarities reinforce the reliability of phenolic profiles as markers for honey characterization. Several of the identified phenolic compounds, including caffeic acid, gallic acid, naringin, and myricetin, are well known for their antibacterial properties (Kachmar et al. 2025). Their presence suggests a potential contribution to the antimicrobial activity of the tested honey samples, which will be explored in the following section.

**TABLE 2:** Concentrations (μg/mg) of phenolic compounds in honey samples.

Molecules	RT (min)	Honey origin			
		Citrus honey	Euphorbia honey	Carob honey	Anise honey
Gallic acid	7.68	0.21	NI	21.02	NI
Catechin	12.0	2.69	NI	3.66	NI
3‐Hydroxy benzoic acid	13.6	NI	NI	NI	76.59
Caffeic acid	13.9	NI	285.35	NI	NI
trans‐ferulic acid	14.8	NI	125.37	33.99	NI
*p*‐coumaric acid	18.6	NI	190.93	NI	NI
Naringin	20.2	NI	NI	17.95	669.84
Myricetin	25.2	NI	NI	63.99	90.08
trans‐cinnamic acid	30.6	NI	NI	2.75	NI
Kaempferol	42.2	NI	NI	NQ	NI
Quercetin	44.5	NI	NQ	NQ	NI

Abbreviations: NI, Not Identified; NQ, Not Quantified.

#### 3.1.2 Total Polyphenol and Flavonoid Quantification

Phytochemical analysis revealed marked variability in the bioactive compound content among the investigated honey samples, highlighting the strong influence of botanical origin on their chemical composition. Statistically significant differences were observed in both TPC and TFC (*p* < 0.0001). Euphorbia honey exhibited the highest levels, reaching 30.61 mg GAE/100 g for TPC and 10.51 mg QE/100 g for TFC, whereas citrus honey showed considerably lower values, with 13.53 mg GAE/100 g and 5.95 mg QE/100 g, respectively (Table 3). These findings suggest that floral source plays a key role in determining the antioxidant‐related phytochemical profile of honey. Similar observations were reported by Pyrzynska and Biesaga (2009), who demonstrated that although phenolic compounds are commonly present in honey, their concentrations vary substantially depending on botanical origin.

**TABLE 3:** Total phenolic and flavonoid contents in the studied honey samples.

Parameters	Honey origin				*F*	*p*
	Citrus honey	Euphorbia honey	Carob honey	Anise honey		
TPC (mg EAG/100 g)	13.55 ± 0.33^a^	30.61 ± 0.49^b^	28.24 ± 0.34^c^	19.29 ± 0.52^d^	1030.56	< 0.0001
TFC (mg EQ/100 g)	5.94 ± 0.17^a^	10.47 ± 0.57^c^	9.44 ± 0.45^bc^	8.33 ± 0.44^b^	60.63	< 0.0001

*Note:* Values are expressed as mean ± SD (*n* = 3). Statistical analysis was performed using one‐way ANOVA followed by Tukey's post hoc test. Different superscript letters (a–d) indicate statistically significant differences between honey samples (*p* < 0.05). Abbreviations: GAE, gallic acid equivalents; QE, quercetin equivalents; TFC, total flavonoid content; TPC, total phenolic content.

### 3.2 Antibacterial Activity

**FIGURE 2:**
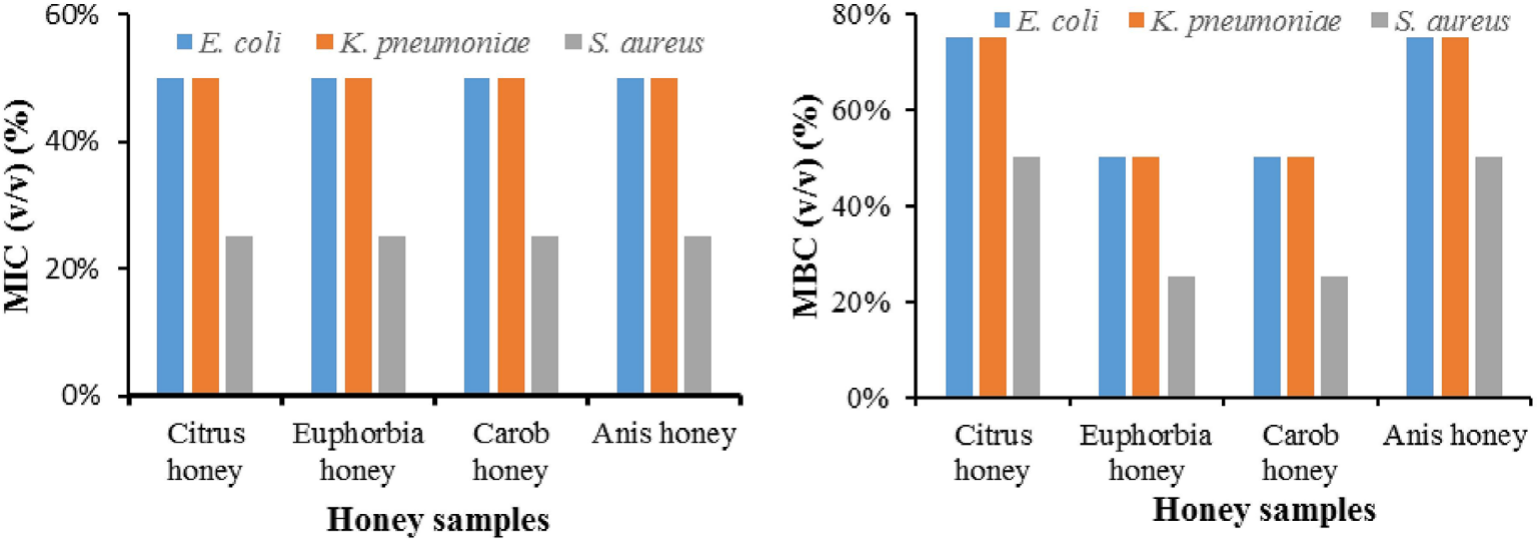
MIC and MBC values of honey samples against *E. coli* , *K. pneumoniae* , and *S. aureus* . Values are expressed as percentage (*v/v*) and represent results obtained from three independent experiments (*n* = 3). MIC and MBC correspond to the lowest honey concentrations required to inhibit visible bacterial growth and to exert a bactericidal effect, respectively.

All tested honey samples exhibited inhibitory activity against the bacterial strains, with MIC values ranging from 25% to 50% (*v*/*v*). Notably, *S. aureus* demonstrated a higher susceptibility, with an MIC of 25% (*v/v*), suggesting a potent antibacterial effect at relatively low concentrations. Conversely, *E. coli* and *K. pneumoniae* required a higher concentration of 50% (*v/v*) for significant bacterial growth inhibition (Figure 2). These results highlight the variability in the inhibitory efficacy of different honey types against the tested bacterial strains. The findings of this study are in accordance with previous research evaluating the antibacterial activity of honey against the same bacterial strains. For instance, a study conducted in Pakistan by Farhana et al. (2023) reported MIC values of 25% and 40% (*w/v*) for *S. aureus* and *E. coli* , respectively. Similarly, in Nigeria, Isa et al. (2020) observed MIC variations in local honey samples, ranging from 20% to 60% (*v/v*) against *S. aureus* , *E. coli* , and *K. pneumoniae* . Conversely, a national study by Belhaj et al. (2016) reported comparatively higher MIC values, reaching 75% (*v/v*) against *E. coli* . Although variations in MIC values exist across studies, these findings collectively support the potential antibacterial efficacy of honey against a broad spectrum of bacterial strains. Moreover, they highlight the influence of key factors such as geographical origin, botanical source, and bacterial strain susceptibility, which may contribute to the observed discrepancies in antibacterial activity.

The MBC values obtained in this study are in agreement with those reported by Mustafa et al. (2022) and Agbabiaka et al. (2020), who observed MBC values ranging from 25% to 75% (*v/v*) for the same bacterial strains. However, these values are lower than those documented by Isa et al. (2020), who reported MBC values between 80% and 100% (*v/v*) against the same pathogens. The discrepancies in MBC values across studies may be attributed to multiple factors, including variations in the botanical origin and composition of honey, differences in methodological approaches for MBC determination, geographical diversity of the honey samples, and potential strain‐specific variations among the tested bacteria.

In our study, the tested honeys demonstrated activity against multidrug‐resistant strains, including *E. coli* resistant to β‐lactams and quinolones, *K. pneumoniae* resistant to β‐lactams and sulfonamides, and *S. aureus* resistant to β‐lactams (Table 4). These findings are consistent with previous studies highlighting the antibacterial properties of honey against multidrug‐resistant pathogenic strains (Agbabiaka et al. 2020; Farhana et al. 2023; McLoone et al. 2020; Owayss et al. 2020; Murugan 2012). While these results highlight honey's antibacterial potential, the high concentrations used (25%–75% v/v) limit direct clinical applicability, and no direct comparison with antibiotics is appropriate.

**TABLE 4:** Antibiotic susceptibility patterns of the tested bacterial strains.

Antibiotics	Dose (μg)	Tested bacteria		
		*E. coli*	*K. pneumoniae*	*S. aureus*
Amikacin	30	S	S	S
Aztreonam	30	S	R	S
Ceftazidime	30	R	R	S
Ciprofloxacin	5	R	S	S
Cefalexin	10	S	S	S
Ceftriaxone	30	R	R	R
Cefotaxime	5	R	R	R
Cefepime	30	S	R	R
Levofloxacin	5	R	S	S
Nalidixic acid	30	R	S	S
Trimethoprim/sulfamethoxazole	25	S	R	S
Ticarcillin + CA	75	R	S	S
Tobramycin	10	R	S	S

Abbreviations: R, resistant; S, sensitive.

The results of our study indicate that the four types of honey analyzed exhibit a broad‐spectrum action, both bacteriostatic and bactericidal, against Gram‐positive and Gram‐negative bacteria, although their efficacy varies depending on the floral origin of the honey and the bacterial strain tested. In this regard, we observed that citrus and anise honeys exhibited the lowest bactericidal activity among the tested honey types. This could be attributed to differences in the floral sources of the honey samples used in this study. Indeed, previous research has shown that the nectar source used in honey production can lead to variations in the antibacterial properties of honeys from different floral origins (Hussein 2000). Furthermore, we also observed that the bacterial strains tested displayed differential sensitivity to the various honey types. Specifically, *S. aureus* (Gram‐positive bacteria) appeared to be more sensitive to the tested honeys compared to *E. coli* and *K. pneumoniae* (Gram‐negative bacteria). These findings are consistent with previous research demonstrating the greater susceptibility of Gram‐positive bacteria compared to Gram‐negative ones (Belhaj et al. 2016; Owayss et al. 2020; Aljaghwani et al. 2021; Cilia et al. 2020). This increased resistance in Gram‐negative bacteria is attributed to their complex three‐layered cell envelope, which provides an additional protective barrier compared to Gram‐positive bacteria (Johnston et al. 2018). Indeed, the antibacterial effect observed in our study could be attributed to the various compounds present in honey, which contribute to reducing or eliminating bacterial activity (Farhana et al. 2023; Mustafa et al. 2022). Among these, phenolic acids and flavonoids are widely recognized as key contributors to honey's antibacterial properties (Kassym et al. 2024). These phenolic compounds, which are secondary metabolites of plants, are introduced into honey during its production, as bees incorporate their enzymatic secretions with floral nectar or plant exudates, both of which contain water, sugars, proteins, and phenolic constituents (Jibril et al. 2019). In the present study, Pearson correlation analysis revealed a highly significant positive relationship between total phenolic content and antibacterial activity (*r* = 0.953), highlighting the substantial role of phenolic compounds in the antimicrobial potential of honey. This association has also been reported in previous studies (Brudzynski 2006; Dżugan et al. 2020).

### 3.3 In Silico Analysis

#### 3.3.1 Molecular Docking

**FIGURE 3:**
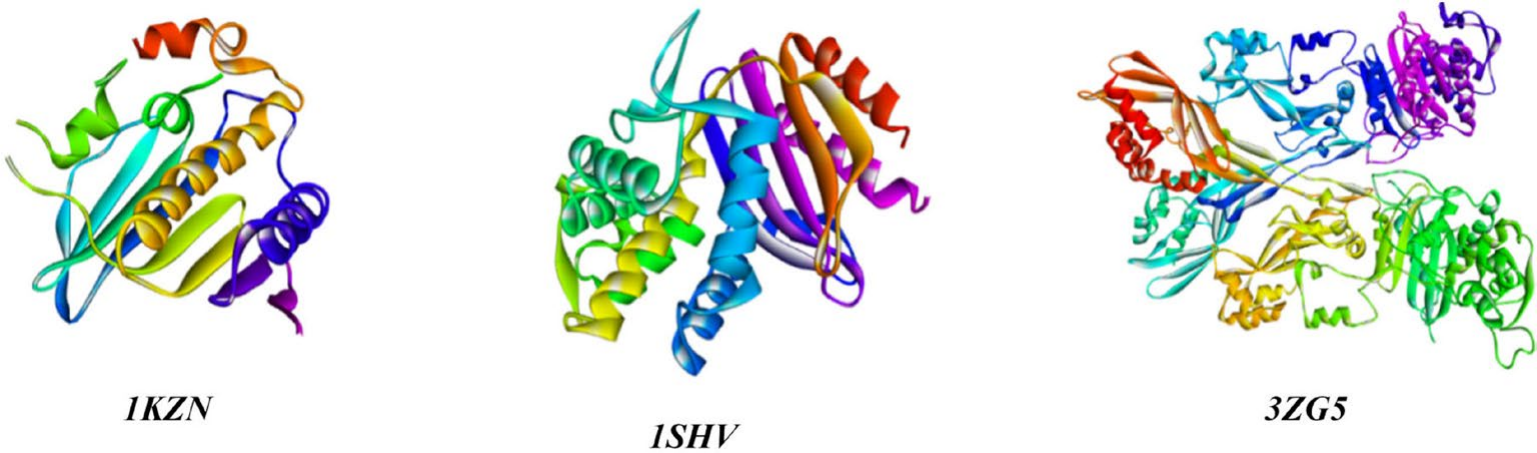
3D ribbon representations of the molecular docking targets: 1KZN (GyrB domain), 1SHV (SHV‐1 β‐lactamase), and 3ZG5 (PBP2a).

Docking analyses centered on three high‐quality crystal structures that represent clinically decisive antimicrobial targets (Figure 3): 1KZN, the 24‐kDa ATP‐binding domain of *E. coli* GyrB whose inhibition blocks bacterial DNA supercoiling; 1SHV, the archetypal SHV‐1 β‐lactamase from *K. pneumoniae* that underlies broad β‐lactam resistance; and 3ZG5, PBP2a from MRSA, the hallmark enzyme conferring methicillin resistance in *S. aureus* (Aati et al. 2025; Zhydzetski et al. 2024). By benchmarking every ligand's binding affinity against ciprofloxacin retained as an internal standard, the resulting score matrix directly gauges each honey‐derived phenolic's ability to compete with a proven antibiotic across three distinct resistance mechanisms. Compounds exhibiting docking energies comparable to those observed for ciprofloxacin across these PDB environments may represent interesting candidates for further investigation against Gram‐negative DNA replication, β‐lactamase‐mediated hydrolysis, or altered MRSA transpeptidase activity. Docking energies (Table 5) reveal four honey phenolics: catechin (M_2_), myricetin (M_8_), kaempferol (M_10_), and quercetin (M_11_) that displayed predicted binding affinities within a range comparable to those observed for ciprofloxacin (−7.7, −5.7, −7.5 kcal/mol for 1KZN, 1SHV and 3ZG5, respectively). Quercetin provides the deepest pose in the GyrB site (−8.3 kcal/mol), while kaempferol dominates both SHV‐1 (−6.5 kcal mol^−1^) and PBP2a (−8.1 kcal/mol), suggesting broad inhibitory potential across DNA‐gyrase, β‐lactamase, and MRSA transpeptidase targets. By contrast, simple benzoic and cinnamic acids (gallic acid (M_1_), 3‐hydroxybenzoic acid (M_3_), coumaric acid (M_6_), and trans‐cinnamic acid (M_9_)) exhibit modest affinities (−4.7 to −5.9 kcal mol^−1^), and naringin (M_7_) fails to engage the PBP2a pocket (−2.0 kcal/mol), reflecting the steric and polarity limitations anticipated from its ADME profile. Overall, kaempferol (M_10_) emerges as the most versatile binder, with catechin (M_2_), myricetin (M_8_), and quercetin (M_11_) displaying favorable docking profiles across multiple targets, warranting further experimental investigation.

**TABLE 5:** AutoDock Vina predicted binding energies (kcal/mol) of ligands M1–M11 and the reference compound Ciprofloxacin against three molecular targets (1KZN, 1SHV, and 3ZG5).

Molecules	1KZN	1SHV	3ZG5
M_1_	−5.7	−4.7	−5.5
M_2_	−8.0	−6.2	−7.7
M_3_	−5.9	−4.8	−5.4
M_4_	−6.3	−5.4	−5.8
M_5_	−6.1	−5.3	−5.8
M_6_	−5.7	−5.4	−5.4
M_7_	−6.4	−5.6	−2.0
M_8_	−8.0	−6.3	−7.8
M_9_	−5.9	−5.8	−5.4
M_10_	−7.9	−6.5	−8.1
M_11_	−8.3	−6.3	−7.6
Ciprofloxacin	−7.7	−5.7	−7.5

*Note:* Lower binding‐energy values indicate stronger predicted ligand–target interactions.

Abbreviations: 1KZN, GyrB domain; 1SHV, SHV‐1 β‐lactamase; 3ZG5, PBP2a.

**FIGURE 4:**
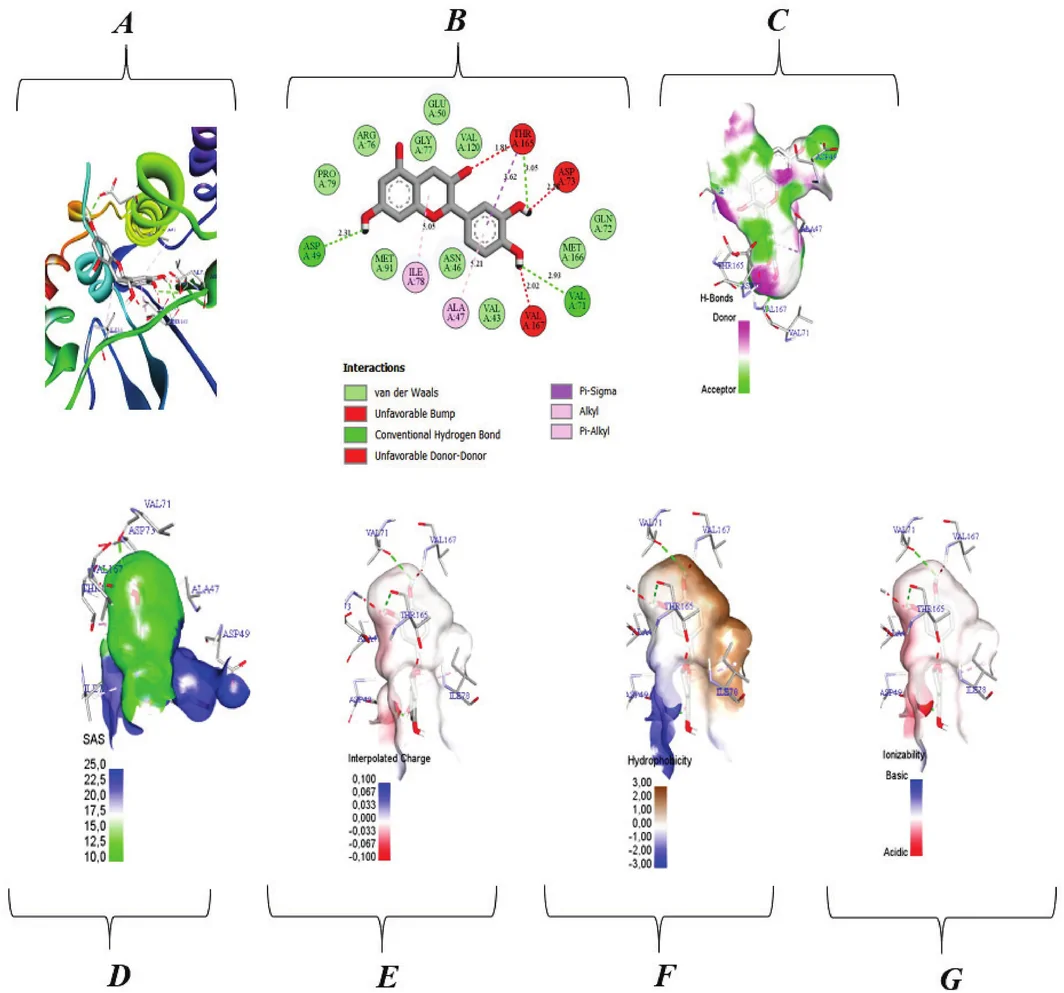
Layered interaction profile of the catechin–GyrB (PDB: 1KZN) complex. (A) Three‐dimensional binding pose within the ATP‐binding pocket; (B) Two‐dimensional interaction diagram highlighting key molecular contacts; (C) Hydrogen‐bond donor/acceptor surface; (D) Solvent‐accessible surface (SAS); (E) Interpolated electrostatic potential map; (F) Hydrophobicity distribution; and (G) Ionizability profile.

Given its balanced ADME profile, minimal predicted toxicity and one of the strongest docking scores in the set, catechin (M_2_) emerges as the most promising honey metabolite. Accordingly, the forthcoming discussion focuses on the catechin‐1KZN complex, examining its binding stability and key intermolecular contacts within the GyrB pocket as a prelude to Figure 4. Ligand catechin (M_2_) nestles deeply inside the ATP‐binding pocket of *E. coli* GyrB (PDB 1KZN), where three tight hydrogen bonds to Asp49 (2.31 Å), Val71 (2.93 Å) and Thr165 (3.05 Å) pin its flavonoid scaffold against both the catalytic rim and the floor of the cleft (Zhydzetski et al. 2024). Ten further van der Waals contacts weave an intimate lattice around the ligand. In contrast, a π–sigma interaction with Thr165 (3.62 Å) and two π‐alkyl contacts at 5.05 Å and 5.21 Å create an aromatic cradle that locks the molecule in place. Surface‐property maps corroborate this picture: the hydrogen‐bond donor/acceptor surface shows a green acceptor belt encircling catechin's hydroxyl donors and magenta donor patches that complement its carbonyl acceptors; the solvent‐accessible surface reveals that most of the ligand is buried, maximizing entropic gain; interpolated charge highlights a negative patch around Asp49/Asp73 that dovetails with catechin's polar groups, whereas the hydrophobic map traces brown streaks under the aromatic rings that coincide with the π‐alkyl contacts; finally, ionizability surfaces display adjacent basic (blue) and acidic (red) regions bridged by the ligand, achieving electrostatic balance. Although three unfavorable donor–donor bumps appear at Thr165 (1.81 Å), Asp73 (2.18 Å) and Val167 (2.02 Å), they sit on the periphery and are unlikely to offset the dominant favorable forces. The combined network of polar, hydrophobic and electrostatically complementary interactions underscored by the ten van der Waals contacts and supportive surface characteristics explains the markedly negative docking energy and underlines the capacity of catechin to obstruct ATP hydrolysis by GyrB, highlighting its promise as a potent antibacterial agent.

#### 3.3.2 Molecular Dynamics Simulation

Molecular dynamics (MD) extends the single docking snapshot by capturing, on the nanosecond scale, the real “breathing” of a protein‐ligand complex. Two main descriptors are extracted: the root‐mean‐square deviation (RMSD), which measures the average quadratic deviation of a set of atoms from a reference structure to assess convergence and stability, and the root‐mean‐square fluctuation (RMSF), which gauges the local motions of each residue to pinpoint flexible versus rigid regions (Hamami et al. 2022).

**FIGURE 5:**
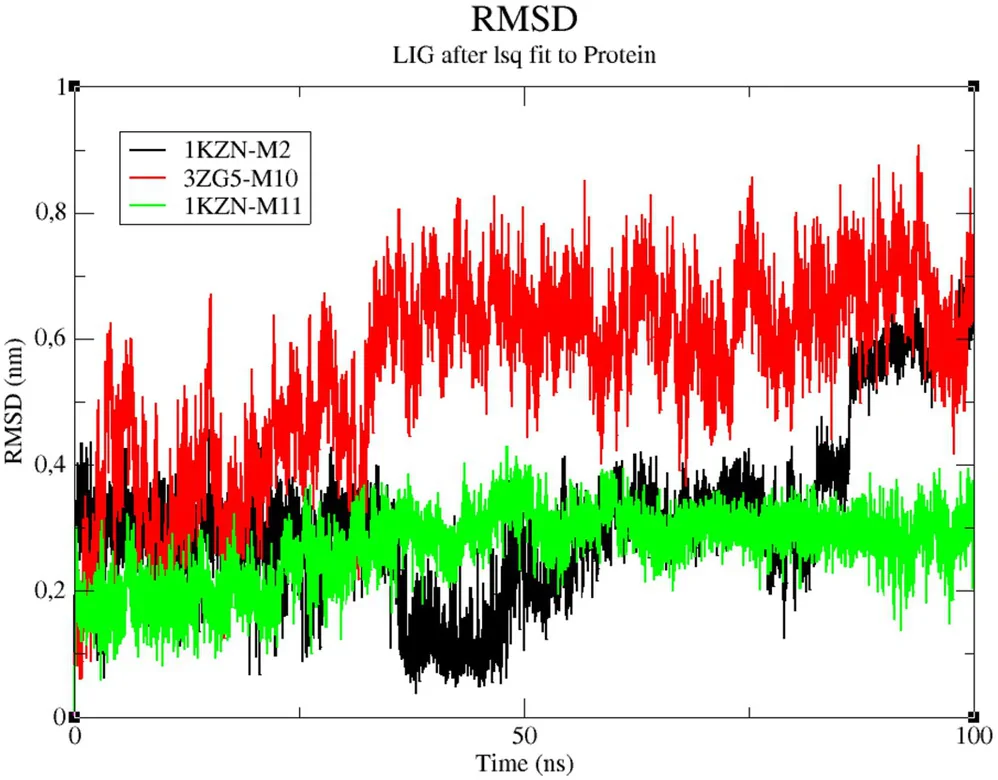
RMSD trajectories over a 100‐ns molecular dynamics simulation for catechin (M2), kaempferol (M10), and quercetin (M11) after least‐squares fitting to their respective target proteins ( *E. coli* GyrB, PDB: 1KZN, and MRSA PBP2a, PDB: 3ZG5).

In Figure 5, 100‐ns ligand RMSD profiles reveal distinct behaviors: quercetin in 1KZN‐M11 (green curve) rapidly plateaus at 0.30–0.35 nm, indicating a firmly anchored pose in the GyrB ATP pocket; catechin in 1KZN‐M2 (black) drops to ~0.15 nm before gradually rising to oscillate near 0.60 nm after 70 ns, suggesting a late readjustment and intermediate stability; kaempferol in 3ZG5‐M10 (red) quickly climbs to ~0.60–0.85 nm and remains highly fluctuating, pointing to a less tightly locked pose in the PBP2a transpeptidase cleft. Thus, the lowest and most consistent RMSD confirms the dynamic superiority of the quercetin‐GyrB complex. At the same time, the other two compounds exhibit greater mobility that could be clarified by targeted RMSF analysis.

**FIGURE 6:**
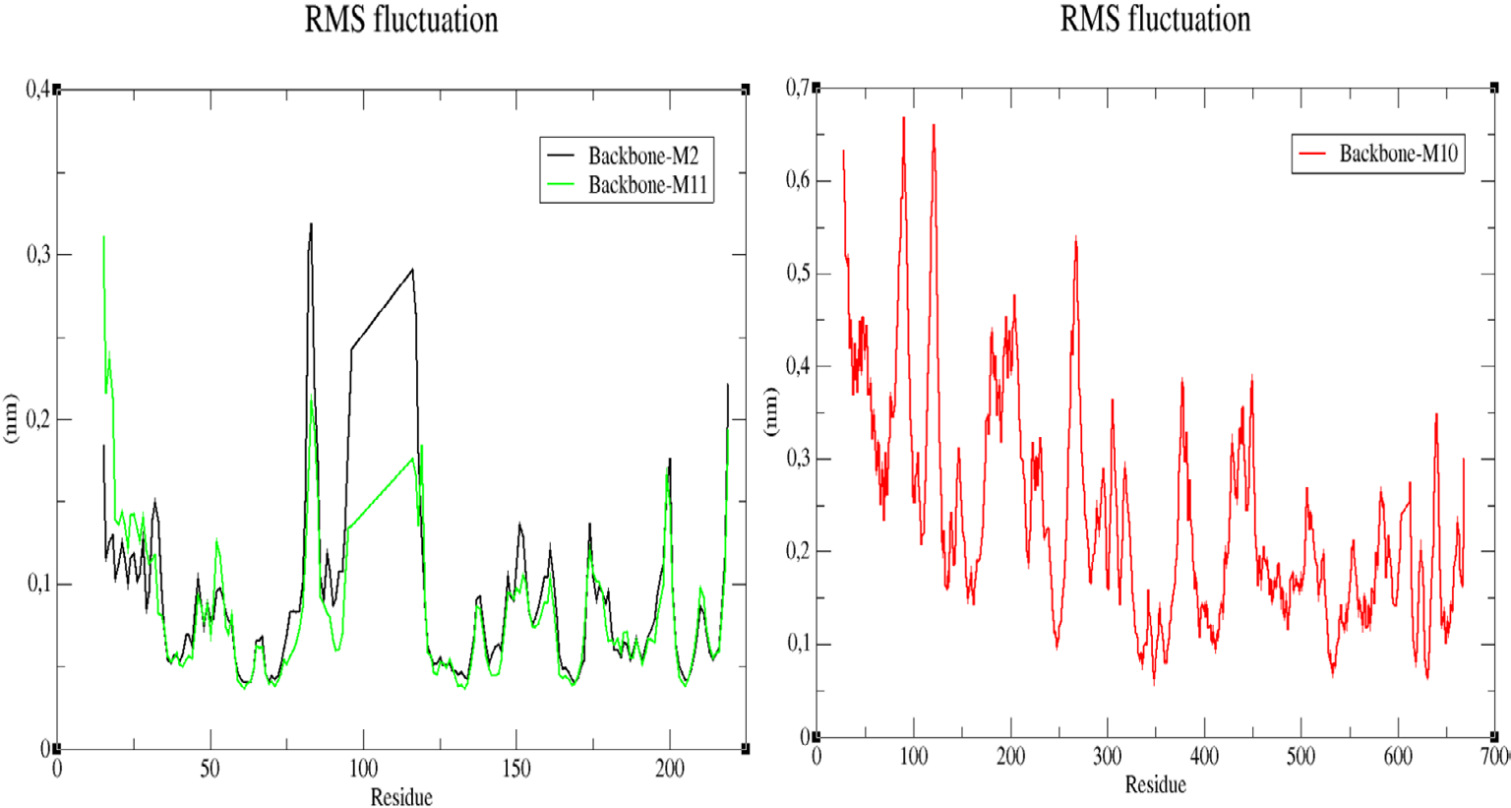
Backbone RMSF profiles over 100 ns for the GyrB‐catechin (M2, black) and GyrB‐quercetin (M11, green) complexes, and for the PBP2a‐kaempferol (M10, red) complex.

Figure 6 plots per‐residue backbone RMSF for the two GyrB complexes (catechin—black, quercetin—green) and the PBP2a complex (kaempferol—red). For GyrB, both ligands keep the catalytic rim (Ala47‐Asp49), the cleft floor (Ile78), and the β‐hairpin that carries Thr165 well below 0.12 nm, confirming that the hydrogen‐bond/π‐stack triad identified in docking remains rigid throughout the trajectory. The only large excursion (≈0.30 nm at residues 90–110) lies in a solvent‐exposed loop far from the binding site and does not disrupt the pocket. Notably, quercetin slightly damps fluctuations around residue 165 compared with catechin, mirroring its lower RMSD and tighter anchoring. In PBP2a, the kaempferol trace displays higher overall mobility (up to 0.65 nm); yet the contact belt discussed earlier Lys148‐Ser149, Thr165/Val165 region, Arg151, Val256, Arg241 and Met372 stays under 0.25 nm, whereas adjacent loops rise well above that value. This differential rigidity suggests that the π‐σ, π‐alkyl and quadruple H‐bond network stabilizes the transpeptidase cleft even as peripheral regions flex freely. Taken together, the RMSF patterns in Figure 6 corroborate the docking‐derived interaction maps: residues directly engaged by the three flavonoids remain dynamically restrained, underscoring the persistence and specificity of their binding contacts over the 100‐ns simulations.

#### 3.3.3 SwissADME In Silico Profiling and BOILED‐Egg Analysis

In this in silico screening, we focus on eleven phenolic metabolites M1: gallic acid, M2: catechin, M3: 3‐hydroxybenzoic acid, M4: caffeic acid, M5: trans‐ferulic acid, M6: p‐coumaric acid, M7: naringin, M8: myricetin, M9: trans‐cinnamic acid, M10: kaempferol, and M11: quercetin, identified as dominant constituents of the four honey samples and whose concentrations correlate positively with the observed bactericidal activity against *E. coli, K. pneumoniae*, and *S. aureus* . To benchmark binding energies and pharmacological relevance, we added ciprofloxacin (M12) as a clinical standard.

Table 6 shows that the majority of the honey‐derived phenolics (gallic acid (M_1_), p‐coumaric acid (M_6_), trans‐cinnamic acid (M_9_), and quercetin (M_11_)) occupy a physicochemical window largely compatible with oral drugs: molecular weights range from 138 to 302 g/mol (all below ciprofloxacin's 331 g/mol) and cLogP values stay within the optimally balanced 0–2 region, indicating sufficient polarity for solubility without incurring permeability penalties. Their topological polar surface areas (TPSA 37–131 Å^2^) cluster around that of the benchmark (74.6 Å^2^), predicting efficient passive diffusion through the intestinal epithelium, a prediction borne out by the “High” GI‐absorption flag and bioavailability scores ≥ 0.55. By contrast, naringin (M_7_) stands out as an outlier: its extremely high MW (≈581 g/mol), inflated hydrogen‐bond counts (14 acceptors/8 donors), and TPSA of 225 Å^2^ generate three Lipinski and one Veber violation, translating into “Low” absorption and the poorest bioavailability score (0.17) (Trott and Olson 2010). Myricetin (M_8_) also straddles oral‐drug limits (TPSA≈152 Å^2^) and registers dual rule violations, yet retains a moderate cLogP (0.79) that partly offsets its permeability deficit. Skin permeability, approximated by log Kp, follows polarity trends: trans‐cinnamic acid (M_9_) exhibits the least negative value (−5.69 cm s^−1^) and thus the highest dermal penetration potential, whereas M_7_ drops to −10.15 cm s^−1^, more impermeable than ciprofloxacin (−9.09 cm s^−1^). Efflux liability was predicted for catechin (M2), naringin (M7), and ciprofloxacin (M12), all identified as potential P‐gp substrates, suggesting possible transporter‐mediated reductions in intracellular accumulation. The predicted risk of cytochrome‐mediated drug–drug interactions appeared limited for catechin (M2), naringin (M7), and trans‐cinnamic acid (M9). In contrast, gallic acid (M1), myricetin (M8), kaempferol (M10), and quercetin (M11) were predicted to inhibit CYP3A4, while kaempferol (M10) and quercetin (M11) additionally showed potential CYP2D6 inhibition. These predictions may indicate a possible influence on the pharmacokinetics of co‐administered drugs and warrant further experimental investigation. Unlike these compounds, such inhibitory effects were not predicted for ciprofloxacin (Ramadhan et al. 2023).

**TABLE 6:** Key ADME and drug‐likeness parameters for M1‐M11 versus ciprofloxacin (M_12_).

Parameter	M1	M2	M3	M4	M5	M6	M7	M8	M9	M10	M11	Ciprofloxacin
Molecular weight (g/mol)	170.12	290.27	138.12	180.16	194.18	164.16	580.53	318.24	148.16	286.24	302.24	331.34
H‐bond acceptors	5	6	3	4	4	3	14	8	2	6	7	5
H‐bond donors	4	5	2	3	2	2	8	6	1	4	5	2
Rotatable bonds	1	1	1	2	3	2	6	1	2	1	1	3
TPSA (Å^2^)	97.99	110.38	57.53	77.76	66.76	57.53	225.06	151.59	37.3	111.13	131.36	74.57
CLogP	0.21	0.85	1.03	0.93	1.36	1.26	−0.85	0.79	1.79	1.58	1.23	1.1
Lipinski: violations	0	0	0	0	0	0	3	1	0	0	0	0
Veber: violations	0	0	0	0	0	0	1	1	0	0	0	0
GI absorption	High	High	High	High	High	High	Low	Low	High	High	High	High
Bioavailability Score	0.56	0.55	0.85	0.56	0.85	0.85	0.17	0.55	0.85	0.55	0.55	0.55
log Kp (cm/s)	−6.84	−7.82	−6.08	−6.58	−6.41	−6.26	−10.15	−7.4	−5.69	−6.7	−7.05	−9.09
Pgp substrate	No	Yes	No	No	No	No	Yes	No	No	No	No	Yes
CYP2D6 inhibitor	No	No	No	No	No	No	No	No	No	Yes	Yes	No
CYP3A4 inhibitor	Yes	No	No	No	No	No	No	Yes	No	Yes	Yes	No

Abbreviations: CYP, Cytochrome P450; GI, Gastrointestinal absorption; log Kp, coefficient de perméabilité cutanée logarithmique; P‐gp, P‐glycoprotein; TPSA, Topological Polar Surface Area.

**FIGURE 7:**
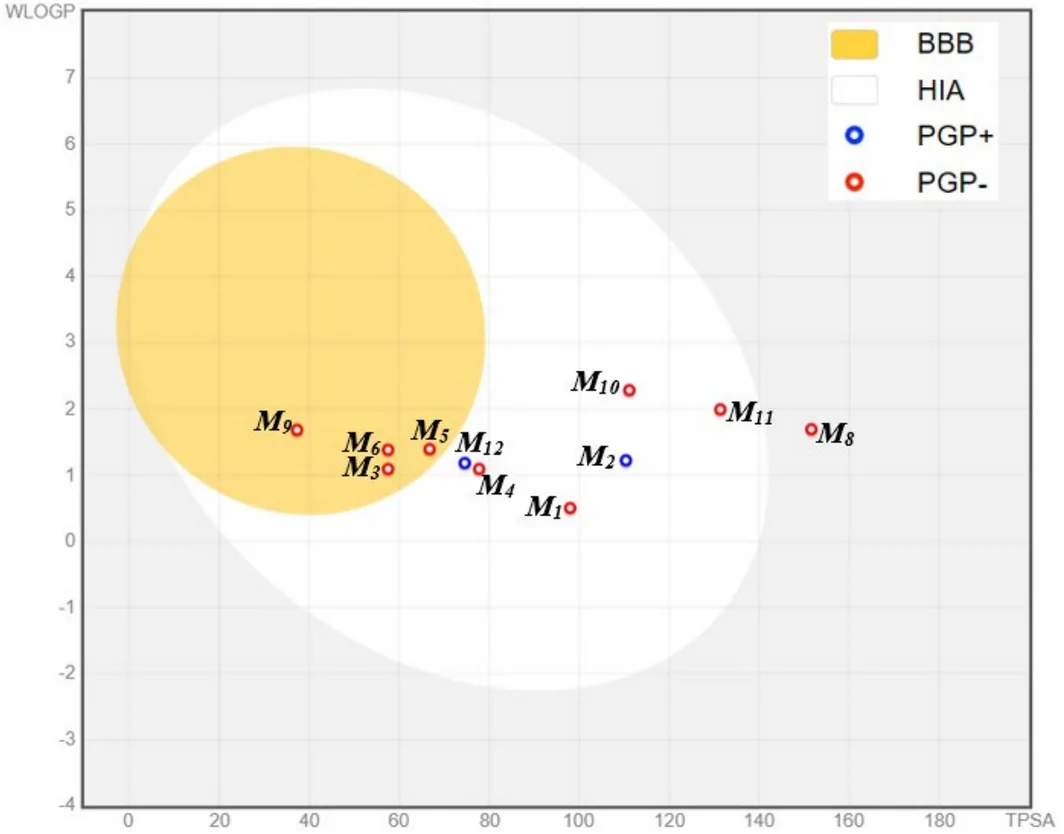
SwissADME BOILED‐Egg plot of *W log P* versus TPSA for honey‐derived ligands M_1_–M_11_ and reference ciprofloxacin (M_12_).

Collectively, the data position 3‐hydroxybenzoic acid (M_3_), trans‐ferulic acid (M_5_), p‐coumaric acid (M_6_) and trans‐cinnamic acid (M_9_) as the phenolics whose rule‐of‐five compliance, high intestinal absorption, lack of transporter or CYP liabilities and favorable bioavailability scores most closely mirror and in some respects surpass the reference profile of ciprofloxacin (M_5_), whereas naringin (M_7_) and, to a lesser extent, myricetin (M_8_) constitute the least orally promising members of the set. In the SwissADME BOILED‐Egg plot (Figure 7) (Ramadhan et al. 2023), W log P is mapped against TPSA to distinguish molecules likely to show high human‐intestinal absorption (white ellipse) from those also able to cross the blood–brain barrier (central yellow core). All ligands appear on the plot except naringin (M_7_), whose extreme polarity (TPSA≈225 Å^2^) places it beyond the frame. Three phenolic acids: 3‐hydroxybenzoic (M_3_), p‐coumaric (M_6_) and trans‐cinnamic acid (M_9_) sit well inside the yellow core, indicating a dual potential for efficient oral uptake and CNS penetration, and they are flagged as non‐substrates for P‐gp (red markers). Ciprofloxacin (M_12_) and trans‐ferulic acid (M_5_) lie on the yellow/white boundary, suggesting good absorption but limited brain entry, while gallic acid (M_1_) and caffeic acid (M_4_) occupy a similar position within the white ellipse. Myricetin (M_8_) is present yet plotted just outside the white region, consistent with its predicted “low” intestinal absorption in Table 6 and negligible CNS accessibility. The more polar flavonoids catechin (M_2_), kaempferol (M_10_) and quercetin (M_11_) cluster near the outer rim of the white area, where rising TPSA curtails permeability; only catechin (M_2_) and ciprofloxacin (M_12_) appear in blue, marking them as likely P‐gp substrates. Overall, the diagram identifies 3‐hydroxybenzoic (M_3_), p‐coumaric (M_6_) and trans‐cinnamic acid (M_9_) as the most pharmacokinetically favorable honey metabolites, whereas the pronounced polarity of myricetin (M_8_) and especially naringin (M_7_) undermines their oral prospects.

#### 3.3.4 ADMETlab 3 Thermal Stability and Pharmacokinetic Profiling

**FIGURE 8:**
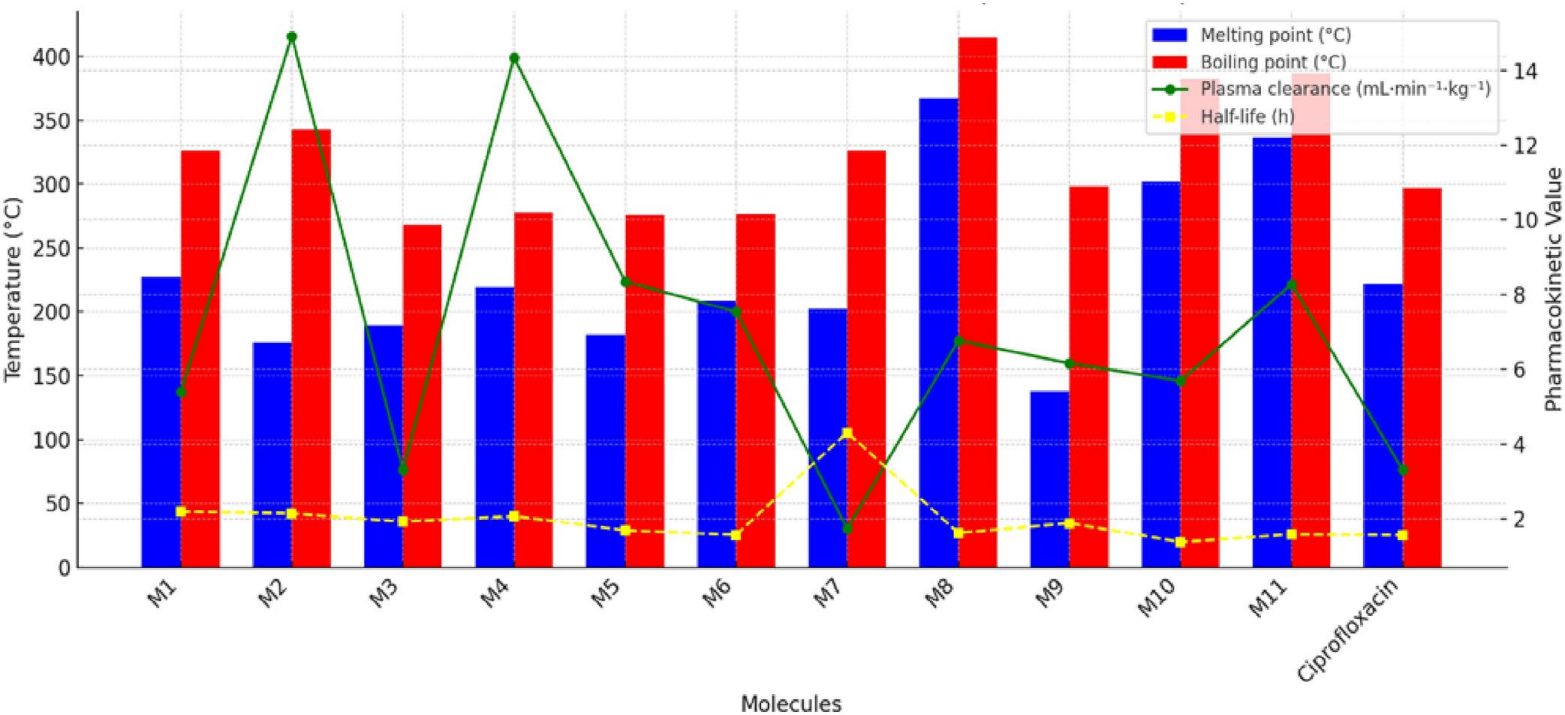
Predicted thermal properties (mp/bp) and pharmacokinetic surrogate (Cl‐t½) from ADMETlab 3 for M1‐M11 versus ciprofloxacin.

ADMETlab 3 data (Figure 8) reveal a wide variation in solid‐state stability: melting‐ and boiling‐point outputs link a compound's thermal endurance during manufacturing to its physical persistence, while the plasma‐clearance/half‐life pair indicates how long it remains systemically active in the body (Daina and Zoete 2016). Myricetin (M_8_) is the most refractory, with a melting/boiling point of 367/415°C, whereas trans‐cinnamic acid (M_9_) is the most volatile at 138/298°C. Ciprofloxacin (M_12_) and the simpler phenolic acids gallic acid (M_1_), 3‐hydroxybenzoic (M_3_), caffeic acid (M_4_), trans‐ferulic acid (M_5_) and p‐coumaric (M_6_) occupy the mid‐range (~180°C–227°C for mp and 268°C–326°C for bp), values compatible with conventional solid‐dosage processing. Pharmacokinetic surrogates show plasma clearance values from 1.73 mL min^−1^ kg^−1^ for naringin (M_7_) up to 14.92 mL min^−1^ kg^−1^ for catechin (M_2_), with half‐lives varying inversely from 4.31 h to about 2 h. The ligand that most closely mirrors ciprofloxacin (Cl = 3.31 mL min^−1^ kg^−1^, t_0.5_ = 1.56 h) is 3‐hydroxybenzoic acid (M_3_), exhibiting an identical clearance, a half‐life of 1.92 h, and a manageable mp/bp of ≈190/268°C. trans‐ferulic (M_5_) and p‐coumaric (M_6_) acids show intermediate clearances (8.35 and 7.54 mL min^−1^ kg^−1^) and half‐lives of 1.69 and 1.57 h, respectively, with acceptable thermal robustness, but align less precisely with the reference profile. Naringin's long half‐life (4.31 h) is offset by its extreme polarity and anticipated permeability limitations, whereas the rapid clearance of catechin (M_2_) and caffeic acid (M_4_) would likely require more frequent dosing to maintain therapeutic levels. In summary, 3‐hydroxybenzoic acid (M_3_) emerges as the only natural candidate that combines suitable thermal stability with a clearance/half‐life balance closely matching ciprofloxacin. trans‐ferulic and p‐coumaric acids (M_5_ and M_6_) remain conditionally promising, while myricetin (M_8_) stands out for exceptional thermal stability but offers only moderate clearance and limited permeability.

#### 3.3.5 In Silico Toxicity Profiling

ProTox‐III predicts a generally moderate acute‐toxicity profile for the honey phenolics (Table 7): gallic acid (M_1_), 3‐hydroxybenzoic acid (M_3_), caffeic acid (M_4_), trans‐ferulic acid (M_5_), p‐coumaric acid (M_6_), naringin (M_7_), trans‐cinnamic acid (M_9_), kaempferol (M_10_), and ciprofloxacin (M_12_) fall into GHS classes 4–5 with oral LD_50_ values of 1772–3910 mg/kg (Ciocarlan et al. 2024), whereas catechin (M_2_) shows the widest safety margin in class 6 (≈10,000 mg/kg). Myricetin (M_8_) and quercetin (M_11_) are more worrisome, both assigned to class 3 (LD_50_≈159 mg/kg). Hepatotoxicity is absent across the panel except for a single “active” call in trans‐cinnamic acid (M_9_) with a 54% probability (Table 7). Carcinogenicity is flagged for gallic acid (M_1_), caffeic acid (M_4_), p‐coumaric acid (M_6_), and myricetin (M_8_) with probabilities ranging from 50% to 78% (Table 7), while immunotoxicity is activated only in trans‐ferulic acid (M_5_), naringin (M_7_), and trans‐cinnamic acid (M_9_) at very high confidence (91%–99%) (Table 7). Mutagenicity remains largely silent, apart from a low‐probability activation in myricetin (51%) and a moderate signal in ciprofloxacin (75%) (Table 7). Cytotoxicity is predicted inactive for all compounds, including the reference drug. Overall, catechin (M_2_) retains the cleanest safety profile, whereas quercetin (M_11_) and especially myricetin (M_8_) accumulate multiple toxicity alerts that merit thorough experimental follow‐up (Table 7).

**TABLE 7:** ProTox‐III prediction of acute toxicity (GHS class and oral LD_50_) and toxicity endpoint assessment for ligands M1–M11 and ciprofloxacin (M12).

Parameter	M_1_	M_2_	M_3_	M_4_	M_5_	M_6_	M_7_	M_8_	M_9_	M_10_	M_11_	Ciprofloxacin
LD_5 0_ (mg/kg)	2000	10,000	2000	2980	1772	2850	2300	159	2500	3919	159	2000
Class	4	6	4	5	4	5	5	3	5	5	3	5
Hepatotoxicity	Ina	Ina	Ina	Ina	Ina	Ina	Ina	Ina	A	Ina	Ina	Ina
Probability (%)	61	72	52	57	51	51	81	69	54	68	69	65
Carcinogenicity	A	Ina	Ina	A	Ina	A	Ina	A	Ina	Ina	Ina	Ina
Probability (%)	56	51	51	78	61	50	90	68	82	72	68	57
Immunotoxicity	Ina	Ina	Ina	Ina	A	Ina	A	Ina	A	Ina	Ina	Ina
Probability (%)	99	96	99	50	91	91	99	86	95	96	87	91
Mutagenicity	Ina	Ina	Ina	Ina	Ina	Ina	Ina	A	Ina	Ina	Ina	A
Probability (%)	94	55	99	98	96	93	73	51	96	52	51	75
Cytotoxicity	Ina	Ina	Ina	Ina	Ina	Ina	Ina	Ina	Ina	Ina	Ina	Ina
Probability (%)	91	84	86	86	88	81	66	99	83	98	99	92

Abbreviations: A, Active; GHS, Globally Harmonized System of Classification and Labelling of Chemicals; Ina, Inactive; LD_50_, median lethal dose.

### 3.4 Limitations of the Study

This study has several limitations. It was limited to four honey samples from specific sites, which may not fully represent the diversity of honeys in the Beni Mellal‐Khenifra region. The botanical origin of the honeys was not confirmed through pollen analysis; instead, monofloral honeys were identified using a controlled field collection strategy, which, while commonly used in beekeeping, may be less precise than microscopic pollen examination. The number of bacterial isolates tested was also limited, which may affect the generalizability of the antibacterial findings. The concentrations of honey tested (25%–75% v/v) are relatively high and may not be achievable in vivo, so the results primarily reflect the antibacterial potential of honey rather than its direct clinical efficacy. Additionally, the in silico analyses presented are hypothesis‐generating and have not been experimentally validated. These limitations highlight the need for further research using physiologically relevant concentrations, larger panels of bacterial isolates, and in vivo models to better assess the therapeutic potential of honey against bacterial pathogens.

## 4 Conclusion

The results of this study confirm the therapeutic potential of monofloral honeys from the Beni Mellal‐Khenifra region, which are rich in phenolic compounds, both as natural antibacterial agents and as sources of bioactive metabolites with pharmacological interest. These honeys demonstrated marked efficacy against multidrug‐resistant uropathogenic strains. The in silico analyses further reinforced these findings by revealing that specific phenolic compounds, such as catechin, myricetin, kaempferol, and quercetin, exhibit strong binding affinities to key bacterial targets including GyrB, SHV‐1 β‐lactamase, and PBP2a. Moreover, ADME and toxicological profiling identified catechin and 3‐hydroxybenzoic acid as the most promising candidates due to their good absorption, low predicted toxicity, and compatibility with oral administration. These data support the relevance of further research aimed at isolating, purifying, and evaluating these compounds in vivo, with the goal of developing clinically exploitable formulations.

## Author Contributions

**Mohammed Al‐Zharani:** funding acquisition, resources, visualization. **Aziz Galman:** software, investigation, data curation, writing – original draft. **Fahd A. Nasr:** funding acquisition, validation, resources, writing – original draft. **Toufik Bouddine:** resources. **Mohamed Reda Kachmar:** methodology, writing – original draft, writing – review and editing. **Rachid Lotfi:** formal analysis, methodology, writing – original draft, writing – review and editing. **Mourad Chikhaoui:** conceptualization, methodology, writing – original draft, formal analysis, software. **Ashraf Ahmed Qurtam:** funding acquisition, validation, resources, writing – original draft, writing – review and editing. **Ahmed Chetoui:** formal analysis, investigation, writing – original draft. **Houda El Hajjouji:** conceptualization, writing – original draft, validation, supervision, writing – review and editing. **Oussama Khibech:** writing – original draft. **Mohamed Bouhrim:** visualization, software, validation, formal analysis, writing – original draft, writing – review and editing, supervision.

## Funding

This work was supported and funded by the Deanship of Scientific Research at Imam Mohammad Ibn Saud Islamic University (IMSIU) (grant number IMSIU‐DDRSP2601).

## Consent

The authors have nothing to report.

## Conflicts of Interest

The authors declare no conflicts of interest.

## Data Availability

The data presented in this study are available on request from the corresponding author.
